# Crise vaso-occlusive drépanocytaire sévère: aspects cliniques, étiologiques et thérapeutiques au CHU Sylvanus Olympio de Lomé

**DOI:** 10.11604/pamj.2024.47.162.33754

**Published:** 2024-04-03

**Authors:** Koffi Mawuse Guedenon, Djatougbe Ayaovi Elie Akolly, Mawouto Fiawoo, Fidèle Comlan Dossou, Ounoo Elom Takassi, Koffi Edem Djadou, Yawo Dzayissé Atakouma, Adama Dodji Gbadoe

**Affiliations:** 1 Département de Pédiatrie, CHU Sylvanus Olympio, Faculté des Sciences de la Santé, Université de Lomé, Lomé, Togo

**Keywords:** CVO sévères, drépanocytaire, étiologies, infections bactériennes, Lomé, Severe VOC, sickle cell disease, bacterial infections, Lomé

## Abstract

**Introduction:**

la crise vaso-occlusive (CVO) est la plus fréquente manifestation de la drépanocytose et la première cause d´hospitalisation des enfants atteints. L´objectif de cette étude est de décrire les aspects cliniques des CVO sévères, de déterminer les étiologies des syndromes infectieux qui les accompagnent et de décrire leur prise en charge.

**Méthodes:**

il s'agit d'une étude transversale descriptive portant sur 137 drépanocytaires majeurs hospitalisés pour CVO sévères du 1^er^ janvier 2009 au 31 décembre 2011 dans le service de pédiatrie du CHU Sylvanus Olympio.

**Résultats:**

les drépanocytaires homozygotes SS étaient les plus nombreux (n=98; 71,5%), suivis des doubles hétérozygotes SC (n=28; 20,5). Le délai moyen de consultation était de 4,7 ± 4,4 jours. Le traitement avant l´admission comportait des antibiotiques (28,5%). Les CVO étaient surtout ostéo-articulaires (70,8%). Dans 98,5% des cas, une infection bactérienne associée a été confirmée (48,9%) ou présumée (49,6%). Les principales étiologies étaient le syndrome thoracique aigu (26,3%), l´ostéomyélite aiguë (10,9%), l´infection urinaire (6,6%), la septicémie (3,6%). Un germe a été isolé chez 14,6% des patients, Escherichia coli (30%) étaient en tête suivi de Klebsiella pneumoniae (25%), Staphylococcus aureus (15%), Salmonella typhi (10%), Streptococcus pneumoniae (5%), le Streptocoque D (5%), l´Enterobacter (5%) et l´Acinetobacter (5%). Le taux de mortalité était de 2,2%. La durée moyenne d´hospitalisation était de 11,4 ± 8,8 jours.

**Conclusion:**

les CVO drépanocytaires sévères sont en majorité associées aux infections bactériennes en milieu tropical. Une antibiothérapie adaptée et précoce constitue le moyen thérapeutique indispensable pour prévenir ou traiter ces patients.

## Abstract (en)

**Introduction:**

vaso-occlusive crisis (VOC) is the most common manifestation of sickle cell disease and the leading cause of hospitalization among affected children. The purpose of this study is to describe the clinical features of severe VOCs, to determine the etiologies of infectious syndromes that accompany them and to describe their management.

**Methods:**

we conducted a descriptive cross-sectional study of 137 adult patients with sickle cell disease hospitalised for severe VOC in the Paediatric Department of the Sylvanus Olympio University Hospital from 1^st^ January 2009 to 31^st^ December 2011.

**Results:**

the majority of patients (n=98; 71.5%) had homozygous sickle cell (SS), followed by double heterozygous SC disease (n=28; 20.5). The median of consultation time was 4.7 ± 4.4 days. Treatment before admission was based on antibiotics (28.5%). VOCs were mainly osteoarticular (70.8%). In 98.5% of cases, an associated bacterial infection was confirmed (48.9%) or suspected (49.6%). The main etiologies included acute chest syndrome (26.3%), acute osteomyelitis (10.9%), urinary tract infection (6.6%) and septicaemia (3.6%). One germ was isolated from 14.6% of patients: Escherichia coli (30%), followed by Klebsiella pneumoniae (25%), Staphylococcus aureus (15%), Salmonella typhi (10%), Streptococcus pneumoniae (5%), Streptococcus D (5%), Enterobacter (5%) and Acinetobacter (5%). Mortality rate was 2.2%. The average length of stay in hospital was 11.4 ± 8.8 days.

**Conclusion:**

severe sickle cell-related vaso-occlusive crisis is mainly associated with bacterial infections in tropical environments. Appropriate and early antibiotic therapy is the essential therapeutic means to prevent or treat these patients.

## Introduction

La drépanocytose est la maladie génétique la plus répandue dans la région africaine de l´OMS [1]. Sa prévalence est estimée entre 10 et 40% en Afrique [2]. Au Togo, cette prévalence est de 16% toutes formes confondues avec 2 à 3% de formes majeures [3]. Elle est à l´origine de l´équivalent de 5% des décès d´enfants de moins de cinq ans sur le continent africain [4]. Depuis mai 2005, l´OMS l´a ajoutée sur la liste des priorités de santé publique [4]. La crise vaso-occlusive (CVO) est sa plus fréquente manifestation clinique [5] et la première cause d´hospitalisation de l´enfant drépanocytaire [6,7]. Le nombre de crises détermine la gravité de la maladie [8]. L´intensité de la CVO est variable. On distingue la CVO commune d´intensité moindre, prise en charge le plus souvent à domicile et la CVO sévère qui nécessite une consultation à l´hôpital. Plus de 1500 patients drépanocytaires majeures sont suivis en ambulatoire dans l´unité d´hématologie oncologie du service de pédiatrie du CHU Sylvanus Olympio (CHU S.O.). Au Togo, 70% des drépanocytaires hospitalisés souffrent de douleurs ostéo-articulaires. La moindre hésitation à l´admission de ces patients est préjudiciable d´une augmentation de la mortalité. Au Togo, on dispose de très peu de données sur les CVO. C´est pourquoi nous avons réalisé ce travail dont l´objectif était de décrire les aspects cliniques, étiologiques, thérapeutiques et évolutifs des CVO sévères.

## Méthodes

**Cadre d´étude**: cette étude a été initiée dans l´unité d´hématologie oncologie (P1) du service de pédiatrie du CHU S.O. de Lomé et s´est déroulée dans le pavillon de soins continus P2. Le service a une capacité d´accueil de 170 lits et berceaux répartis dans 10 pavillons d´hospitalisation dont 02 pavillons de soins continus (P2 et P8). C´est le service de référence en matière de santé de l´enfant au Togo. Il est situé au niveau central dans la hiérarchie des structures sanitaires du pays. Chaque année, il accueille environ neuf mille enfants de 0 à 18 ans, dont le tiers est hospitalisé. L´unité d´hématologie oncologie comporte une sous-unité d´oncologie et une sous-unité d´hématologie abritant la section de prise en charge de la drépanocytose.

**Description de la section de prise en charge de la drépanocytose**: environ 1500 enfants drépanocytaires majeurs y sont suivis depuis 1996. Une quarantaine d´entre eux sont admis en consultation de suivi les mercredis après-midi. En cas de CVO sévères, ils sont hospitalisés au P2.

**Description du pavillon de soins continus P2**: le P2 est réservé aux enfants âgés de 1 à 18 ans en détresse vitale. Il a une capacité de 19 lits. Le taux d´occupation des lits est estimé entre 90 et 100%. Le matériel de soins disponible est constitué de deux aspirateurs de mucosité, 4 bouteilles d´oxygène, des bouillottes, une cloche de Hood pour oxygénothérapie, un insufflateur, un tensiomètre pédiatrique, une fiche de température pour chaque patient. Des gestes de réanimation n´y sont pas couramment pratiqués. Quelques rares fois une intubation est faite pour aspirer. Une ventilation à l´ambu sans disposer de ventilateur pour les situations initialement évaluées comme réversibles. En cas d´arrêt cardiaque, les patients bénéficient d´une ventilation à l´ambu associée à un massage cardiaque externe. La drogue disponible dans ces cas est l´épinéphrine qui est utilisé sans un protocole de soins préalablement établi. Notre étude a porté sur 137 patients drépanocytaires qui ont été hospitalisés de manière consécutive pour CVO sévère dans le pavillon P2. Chaque patient hospitalisé avait un dossier d´hospitalisation.

**Les critères d´inclusion**: les patients inclus étaient des drépanocytaires majeurs (SS, SC, Sßthal, SF, DC), ayant tous réalisé une électrophorèse, admis pour une CVO dont l´intensité de la douleur a nécessité une hospitalisation.

**Les critères de non-inclusion**: les drépanocytaires admis pour un syndrome infectieux sans douleur, un priapisme, d´autres complications aiguës ou dégénératives de la drépanocytose sans douleur n´étaient pas été inclus.

**Schéma d´étude**: nous avons réalisé une étude transversale descriptive visant à étudier les aspects épidémiologiques, cliniques, étiologiques, thérapeutiques et évolutifs de la CVO sévère chez les patients drépanocytaires qui étaient hospitalisés du 1^er^ janvier 2009 au 31 décembre 2011 (3 ans).

**Définitions adoptées et paramètres étudiés**: le CVO était un épisode douloureux aigu de durée variable. Les types de CVO étaient le syndrome pieds-mains, la CVO ostéo-articulaire, la CVO thoracique et la CVO abdominale. La CVO mixte portait à la fois sur les os / articulations et l´abdomen. La CVO commune était celle soulagée par un antalgique habituel (palier I ou II), sans signe de gravité et qui n´avait pas nécessité une consultation et traitée en ambulatoire. La CVO sévère était définie comme toute CVO n´ayant pas répondu aux antalgiques habituels et dont la prise en charge avait nécessité une hospitalisation. L´hémoculture était systématique. L´Examen Cytologique et Bactériologique des Urines (ECBU) devrait l´être aussi mais il ne pouvait être réalisé aux heures de garde au CHU Sylvanus Olympio. La septicémie était définie par une hémoculture positive chez un patient présentant des signes cliniques d´une infection généralisée. L´infection urinaire était définie par l´isolement d´une bactérie à la culture des urines avec une bactériurie ≥ 10^5^/mm^3^. Le syndrome thoracique aigu était défini par une toux associée à une douleur thoracique, à une dyspnée et à tout infiltrat pulmonaire radiologique. Le diagnostic de paludisme était retenu sur la base d´une goutte épaisse positive et d´une apyrexie dans les premières 72 heures sous traitement. La CVO sévère d´étiologie indéterminée était retenue si aucun autre diagnostic n´a été retrouvé malgré les examens complémentaires réalisés.

**Description de la méthode d´isolement des bactéries au laboratoire de microbiologie**: les produits pathologiques ont été systématiquement examinés à l´œil nu puis au microscope optique à l´état frais après étalement entre lame et lamelles. On procédait ensuite à la coloration de Gram ou au bleu de méthylène puis la numération bactérienne et on orientait le choix des milieux de culture. L´antibiogramme était réalisé sur le milieu gélosé de type Mueller-Hinton additionné ou non de 5% de sang de mouton (*S. pneumoniae*) et par la méthode des disques. La production de BLSE a été recherchée en plaçant sur la gélose MH le disque de l´amoxicilline + acide clavulanique entre ceux de céfotaxime et ceftazidime. La résistance hétérogène des staphylocoques à toutes les bêta-lactamines a été recherchée en utilisant des disques d´oxacilline ou de céfoxitine. La recherche était positive si la souche était résistante à l´antibiotique après une incubation à 30°C pendant 18 à 24 h. La souche était alors dite méti-R.

**Analyse des données**: les données recueillies ont été saisies dans le logiciel Epi Data. Elles ont été apurées et analysées dans le logiciel SPSS (Statistic Package for Social Science).

## Résultats

**Figure 1:**
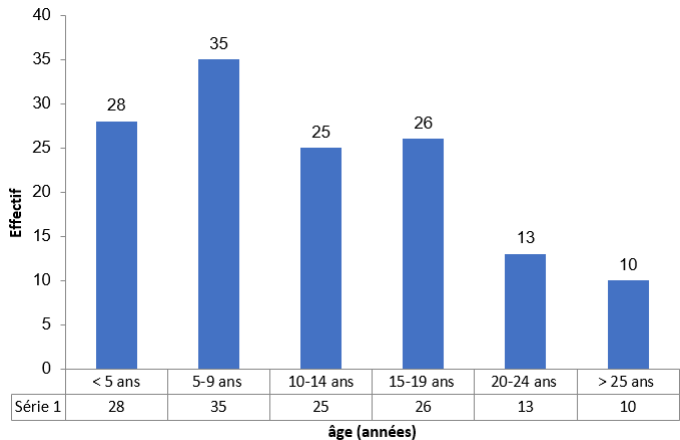
répartition des patients selon l´âge

**Aspects épidémiologiques**: de janvier 2009 à décembre 2011, 13484 patients étaient hospitalisés dans le service de pédiatrie du CHU Sylvanus Olympio dont 4062 au P2. Parmi ces patients, 197 (1,5%) étaient des drépanocytaires. Il y avait 163 cas de CVO sévères (82,7% des drépanocytaires) dont 26 non inclus dans notre étude. Au total, 137 cas étaient retenus. Le nombre moyen de patients hospitalisés par année était de 45,7 ± 16,5 avec des extrêmes de 32 et de 64. L´âge moyen des patients était de 11,7 ± 7,6 ans avec des extrêmes de 1 et 34 ans. Il y avait 114 enfants (83,2%) âgés de 0 à 19 ans dont 28 enfants (20,4%) de moins de 5 ans (Figure 1). Il y avait une prédominance masculine avec un sex-ratio de 1,4.

**Aspects cliniques**

**Répartition des patients selon leurs antécédents**: les drépanocytaires homozygotes SS étaient les plus nombreux (n=98; 71,5%) suivis des drépanocytaires double hétérozygotes SC (n=28; 20,5%). Il y avait 9 patients (6,6%) de profil FS, un patient Sßthal et un patient DC. Trois patients (2,2%) vivaient avec le VIH, 2 patients (1,5%) étaient asthmatiques, un patient (0,7%) était splénectomisé et un patient (0,7%) avait une cardiopathie. Le délai moyen d´évolution de la CVO avant admission était de 4,7 ± 4,4 jours avec un maximum de 21 jours. Sept patients étaient admis dans les 24 premières heures, 101 patients entre le 2^e^ et le 7^e^ jour, et 29 patients après une semaine d´évolution. La majorité des patients (n=115; 83,9%) avait reçu un traitement avant l´admission. Il s´agissait d´un antibiotique chez 28,5% (n=39) avec en tête de liste la ciprofloxacine (n=23; 16,8%).

**Motif de consultation**: la douleur était présente à l´admission chez tous les patients. Son intensité était citée comme motif principal de consultation par tous. Les autres motifs de consultation étaient la persistance de la fièvre (5,8%), la dyspnée/essoufflement (5,8%), le caractère permanent de la douleur (4,4%), la pâleur/anémie (1,5%), une tuméfaction douloureuse du bras (1,5%). La douleur était multifocale dans 44% des cas, unifocale dans 31% et bifocale dans 25% des cas.

**Caractéristiques des CVO**: les CVO ostéo-articulaires étaient le type de CVO le plus fréquent (70,8%), suivi des CVO mixtes (19%), des CVO abdominales (8%) et des syndromes pieds-mains (2,2%).

**Evaluation de la douleur à l´admission**: l´intensité de la douleur était évaluée selon l´échelle visuelle analogique (EVA) 79 fois (57,7%) chez des patients d´âge moyen 15,9 ± 6,5 ans avec des extrêmes de 5 ans et 34 ans. L´intensité de la douleur était supérieure à 5/10 chez tous les patients et 72,2% des patients avaient une douleur d´intensité 10/10 à l´admission.

**Les signes accompagnant la douleur**: la plupart des patients (96,3%) présentaient une fièvre durant leur hospitalisation. Celle-ci n´était pas présente pendant les 24 premières heures d´hospitalisation dans 58,4% des cas. Cinq patients n´avaient pas eu de fièvre. L´asthénie (n=50), la dyspnée (n=43), l´anorexie (n=42), la toux (n=37) et l´impotence fonctionnelle (n=9) étaient les autres signes fonctionnels. Quant aux signes physiques, une douleur osseuse était retrouvée chez 46 patients et un gonflement de segment de membre était noté chez 20 patients. Des signes d´atteintes pulmonaires (râles et souffles) étaient retrouvés chez 33 patients. Il y avait une défense abdominale chez 28 et un œdème péri céphalique chez 6 patients se plaignant de douleur crânienne.

**Aspects étiologiques**: chez la plupart des patients, une infection confirmée ou suspectée était associée à la CVO (Tableau 1). Le syndrome thoracique aigu était bilatéral chez 23 patients et unilatéral chez 13 patients. La goutte épaisse était réalisée chez tous les patients. Elle était positive chez 21 patients dont 19 avaient une infection bactérienne associée. La densité parasitaire moyenne était de 9181,3 ± 20674,3 trophozoïtes/µL avec des extrêmes de 209 et 85052. Les germes isolés dans les bactériémies étaient *Klebsiella pneumoniae* (n=3), *Escherichia coli* (n=1) et *Enterobacter sp* (n=1). L´ECBU était réalisé chez 62 patients. Les germes isolés étaient *E coli* (n=4), *Klebsiella pneumoniae, Staphylococcus aureus*, streptocoque D et *Acinetobacter* chez un patient chacun. Une des souches d´*E coli* isolée dans les urines secrétait une bêta-lactamase à spectre élargi (BLSE).

**Tableau 1:** répartition des patients selon l´étiologie des CVO sévères

	Effectif	Pourcentage (%)
CVO sévère d´étiologie indéterminée	59	43,1
Syndrome thoracique aiguë	36	26,3
Paludisme	21	15,3
Ostéomyélite aiguë	15	10,9
Septicémie	13	9,5
Infection urinaire	9	6,6
Arthrite	3	2,2
Ostéite	1	0,7
Abcès splénique	1	0,7
Abcès sous diaphragmatique et pariétal	1	0,7
Méningite à pneumocoque	1	0,7

**CVO sévère d´étiologie indéterminée**: il y avait une suspicion clinique de septicémie chez 48,9% des patients mais les examens complémentaires n´ont mis en évidence aucune étiologie. Parmi ces patients, 19 avaient une goutte épaisse positive mais le traitement du paludisme avait échoué si bien qu´un diagnostic exclusif de paludisme ne pouvait être retenu. Chez 8 patients, un staphylocoque à coagulase négative était isolé. Deux patients avaient une leucocyturie sans germe. Ces CVO ainsi que les syndromes infectieux qui les accompagnaient évoluaient favorablement sous traitement antibiotique.

**Les germes retrouvés dans les infections responsables de CVO sévères**: un germe était isolé chez 20 patients soit 14,6% (Tableau 2).

**Tableau 2:** répartition des patients selon la fréquence des germes isolés dans les CVO sévères

	Effectif	Pourcentage (%)
Escherichia coli	6	30
Klebsiella pneumoniae	5	25
Staphylococcus aureus	3	15
Salmonella typhi	2	10
Streptococcus pneumoniae	1	5
Streptocoque D	1	5
Enterobacter	1	5
Acinetobacter	1	5

**Aspects thérapeutiques**: la majorité des patients (n=135; 98,5%) avait reçu une antibiothérapie. Une transfusion était faite chez 34,3% des patients (n=47). Les antalgiques étaient utilisés chez la plupart des patients (n=126; 91,9%). Il s´agissait d´antalgique de palier II à savoir la buprénorphine, le tramadol associé au paracétamol injectable. La morphine n´était pas disponible. Les AINS chez 69 patients (50,4%). Il s´agissait du kétoprophène, du diclofénac et de l´ibuprophène. Un antipaludéen était administré chez 73 patients (53,3%). L´oxygène était nécessaire chez 16 patients (11,7%). Le phloroglucinol (antispasmodique) était administré à 13 patients (9,5%). Un plâtre était confiectionné chez 3 patients (2,2%). L´érythropoiétine était administré chez un patient (0,7%). Une arthrotomie était faite chez un patient. Chez 41,5% des patients une bi antibiothérapie était initiée d´emblée. Elle était faite de ceftriaxone + nétilmicine/gentamycine dans 35,6% des cas. Une monothérapie en première intention concernait 58,4% des patients. Il était à base de ceftriaxone (17%), ciprofloxacine comprimé (16,3%), ciprofloxacine injectable (11,1%), amoxicilline acide-clavulanique (5,9%), lévofloxacine (5,9%), vancomycine (1,5%) et clarithromycine (0,7%). La majorité des patients (n=126) avait besoin d´une antibiothérapie de 2e intention (91,9%). Il s´agissait d´un ou de deux antibiotiques ajoutés aux antibiotiques déjà en cours. La ciprofloxacine était utilisée en 2e intention chez 59,5% des patients. La ceftriaxone (18,3%), la clarithromycine (14,3%), la gentamycine (7,1%), la lévofloxacine (7,1%), l´amoxicilline acide-clavulanique (4,8%) et la vancomycine (3,2%) étaient les autres antibiotiques utilisés en deuxième intention. Une antibiothérapie de 3^e^ intention était nécessaire chez 11,7% de patients (n=16). Une antibiothérapie de 4e intention était nécessaire chez 3,6% (n=5) des patients. Deux patients avaient reçu une antibiothérapie de 5^e^ puis de 6^e^ intention. L´artéméther était le principal antipaludique utilisé (n=67; 91,8%). Au cours de leur hospitalisation, un patient était transfusé 3 fois, 4 patients transfusés 2 fois et 42 patients transfusés une fois. Le syndrome thoracique aigu était la pathologie ayant nécessité le plus de transfusion (16 patients sur les 36 cas de STA) suivi des CVO sévères d´étiologie indéterminée (15 sur les 49), des infections urinaires (3 sur les 8 cas) et des septicémies (2 sur les 5 cas).

**Figure 2:**
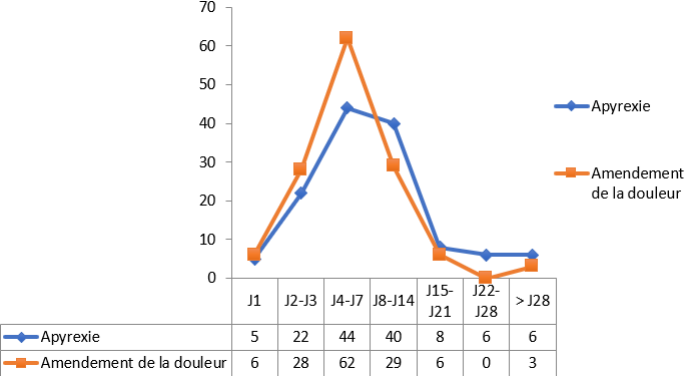
comparaison des délais d´apyrexie et d´amendement de la douleur

**Aspects évolutifs**: le délai moyen d´obtention de l´apyrexie était de 9,8 ± 11,2 jours avec un minimum de 1 jour et un maximum de 90 jours. Quant à la douleur, elle ne s´était pas amendée chez les 3 patients décédés. L´évolution de la fièvre et celle de la douleur étaient superposables (Figure 2). Le taux de létalité était de 2,2% (3 patients décédés). Un des patients décédés avait un STA et les 2 restants avaient un CVO d´étiologie indéterminée. Deux patients décédés avaient entre 15 et 19 ans et le dernier avait plus de 25 ans. Deux patients décédés avaient pris un antibiotique avant admission. Les 3 patients décédés avaient une anémie décompensée et ont été transfusés. Les décès étaient survenus dans un contexte de choc septique. Des 3 patients décédés, 2 étaient drépanocytaires homozygotes SS et un était drépanocytaire composite SC. La majorité des patients (n=132; 96,3%) étaient guéris. Deux patients (1,5%) guéris avaient des séquelles à type de surdité et d´instabilité articulaire. Une ostéomyélite chronique était notée. Une toxidermie type Stevens Johnson compliquait le traitement chez un patient guéri. Deux patients étaient ré hospitalisés. La durée moyenne d´hospitalisation était de 11,4 ± 8,8 jours avec des extrêmes de 2 et 90 jours.

## Discussion

Dans notre méthodologie, la CVO sévère a été définie comme une crise persistante en dépit de l´utilisation des antalgiques habituels à domicile. Dans nos résultats, l´évaluation systématique de la douleur à l´admission a permis de retrouver chez tous les patients une douleur intense. Une douleur d´intensité supérieure ou égale à 5/10 à l´EVA non calmée par un antalgique habituel pourrait définir un CVO sévère et indiquerait une hospitalisation urgente. Le traitement de ces douleurs indique l´utilisation de la morphine ou des dérivés morphiniques dont la non-disponibilité a conduit à une sous-utilisation. Nos patients ont été traités par des antalgiques de palier II et des AINS associés au paracétamol. L´absence de fièvre les premiers jours d´hospitalisation s´expliquerait par le fait que les syndromes infectieux associés aux CVO seraient dus à une surinfection faisant suite à l´obstruction vasculaire. Les infections bactériennes étant ainsi une complication de la CVO sévère. La fréquence de cette complication est si élevée que la CVO sévère ne peut être dissociée des infections bactériennes. Toute dissociation à l´instar d´autres études [8,9] serait tributaire d´une mortalité plus élevée. Dans notre étude, ces deux complications ne pouvaient être dissociées, la CVO sévère pouvant même être considérée comme un symptôme précédant dans la majorité des cas une infection bactérienne en milieu tropical.

La prise en charge adéquate passe donc par une antibiothérapie presque systématique après les prélèvements pour une recherche étiologique. Nos hémocultures ont été positives dans 3,6%. Ce taux était de 1,47% chez Diagne *et al*. au Sénégal [9]. La différence observée peut être liée à l´inclusion dans leurs séries d´autres complications que les CVO. Dans la plupart des pays en Afrique au sud du Sahara, les antibiotiques sont délivrés dans les officines sans prescription médicale. L´automédication à base d´antibiotique observée dans notre série diminue la probabilité d´isoler des germes à l´hémoculture. Ce constat a été également fait par Williams *et al*. à Kilifi au Kenya [10]. Les staphylocoques à coagulase négative retrouvés dans notre série malgré que les prélèvements soient fait dans des conditions d´asepsie rigoureuse ont posé un problème diagnostique car le drépanocytaire n´est pas immunodéprimé et le séjour antérieur en unité de soins intensifs ne suffit pas pour justifier une infection à ce germe. Les autres germes isolés dans notre série demeurent surtout des bacilles à Gram négatif comme dans d´autres études en Afrique subsaharienne mais dans des proportions différentes [9-12]. Classiquement, les salmonelles sont les germes les plus fréquemment isolés dans les ostéomyélites du drépanocytaire [10,13]. Le Staphylocoque à l´instar de notre étude a été le germe le plus rapporté dans les arthrites [14]. L´infection urinaire était une étiologie fréquente des CVO sévères dans notre étude malgré le faible taux de réalisation de l´ECBU. Il en était de même dans d´autres séries [8,14,15]. Il s´agissait d´infection urinaire à bas bruit. Escherichia coli était le germe le plus retrouvé aussi bien dans notre série que dans les autres [15,16].

La prédominance féminine était aussi classique [14,15]. Deux patients (1,4%) ont souffert exclusivement de paludisme, les autres patients ayant une goutte épaisse positive ont été guéri après le traitement antibiotique du foyer infectieux retrouvé. L´impact du paludisme sur le pronostic de la drépanocytose est bien connu [17,18]. La gravité du paludisme chez l´enfant drépanocytaire est bien établie [17,18], cependant dans notre série, le paludisme était rarement responsable d´un CVO sévère et lorsqu´il en était responsable, ces crises étaient rapidement résolutives. Ceci s´expliquerait par les traitements antipaludiques systématiques pris à domicile ou dans les centres périphériques pour enrayer le paludisme à l´étape de paludisme simple. La principale hantise dans notre contexte est la surinfection bactérienne des CVO sévères chez le drépanocytaire en milieu tropical, ceci justifie pour en améliorer le pronostic d´instituer une antibiothérapie adaptée. L´infection a été au centre des préoccupations dans notre étude. Une bi-antibiothérapie a été d´emblée instituée chez 37,9% des patients à cause de la sévérité du tableau clinique à l´admission. L´objectif était d´enrayer le foyer infectieux probable en couvrant les germes souvent retrouvés chez le drépanocytaire. Les infections bactériennes associées aux CVO sévères sont de plus en plus difficiles à traiter. Ceci explique le recours à plusieurs lignes d´antibiotiques et les longs délais d´hospitalisation. C´était l´amendement de la douleur qui signait en général le début de la guérison. Cet amendement de la douleur intervenait souvent au même moment que l´apyrexie comme nous l´avons remarqué dans notre étude. Les décès étaient survenus dans un contexte de choc septique. Les germes responsables de la CVO sévère chez les 2 patients ayant un CVO d´étiologie indéterminée n´ont pas pu être isolés afin d´avoir un antibiogramme adapté pour une prise en charge adéquate. Le traitement avant admission pris a certainement contribué à empêcher l´isolement du germe à l´hémoculture. La guérison de tous les enfants de moins de 5 ans montre l´importance du suivi dans la drépanocytose.

## Conclusion

Les CVO sévères sont les premiers motifs de consultation et d´hospitalisation des drépanocytaires. Ce travail a permis de confirmer le rôle prépondérant des infections bactériennes compliquant les CVO sévères du drépanocytaire. Le paludisme a été rarement mis en cause compte tenu de l´efficacité du traitement systématique des cas. L´antibiothérapie devrait donc être systématique après les prélèvements visant à documenter le germe responsable lors des premiers signes de surinfection des CVO sévères. La fièvre est souvent absente les premiers jours et ne doit surtout pas retarder cette antibiothérapie qui a pour but de prévenir la survenue de cette complication infectieuse.

### Etat des connaissances sur le sujet

- *La fréquence des crises vaso-occlusives sévères dans les services de pédiatrie en Afrique subsaharienne, toujours accompagnées de fièvre;*
- *La difficulté de la prise en charge des patients hospitalisés pour CVO sévère;*
- *Les données n´ont pas changées dans notre pratique clinique quotidienne en dépit du temps écoulé*.

### Contribution de notre étude à la connaissance

- *Les infections bactériennes compliquent toujours les crises vaso-occlusives;*
- *Le Syndrome thoracique aigu, l´ostéomyélite, les infections urinaires et la septicémie sont les étiologies, ceci implique une antibiothérapie adaptée et précoce;*
- *Aucune étude pareille de notre unité, depuis cette étude notre attitude a changé et nous prenons mieux en charge les patients*.
